# ApoE isoform does not influence skeletal muscle regeneration in adult mice

**DOI:** 10.3389/fphys.2023.1302695

**Published:** 2023-11-22

**Authors:** Benjamin I. Burke, Jensen Goh, Fatmah A. Albathi, Taylor R. Valentino, Georgia L. Nolt, Jai K. Joshi, Cory M. Dungan, Lance A. Johnson, Yuan Wen, Ahmed Ismaeel, John J. McCarthy

**Affiliations:** ^1^ Department of Physiology, College of Medicine, University of Kentucky, Lexington, KY, United States; ^2^ Center for Muscle Biology, University of Kentucky, Lexington, KY, United States; ^3^ Buck Institute for Research on Aging, Novato, CA, United States; ^4^ Sanders-Brown Center on Aging, University of Kentucky, Lexington, KY, United States; ^5^ Department of Health, Human Performance, and Recreation, Robbins College of Health and Human Sciences, Baylor University, Waco, TX, United States; ^6^ Division of Biomedical Informatics, Department of Internal Medicine, College of Medicine, University of Kentucky, Lexington, KY, United States

**Keywords:** APOE, regeneration, satellite cells, MuSCs, myogenesis, proliferation, differentiation, metabolism

## Abstract

**Introduction:** Apolipoprotein E (ApoE) has been shown to be necessary for proper skeletal muscle regeneration. Consistent with this finding, single-cell RNA-sequencing analyses of skeletal muscle stem cells (MuSCs) revealed that *Apoe* is a top marker of quiescent MuSCs that is downregulated upon activation. The purpose of this study was to determine if muscle regeneration is altered in mice which harbor one of the three common human ApoE isoforms, referred to as ApoE2, E3 and E4.

**Methods:** Histomorphometric analyses were employed to assess muscle regeneration in ApoE2, E3, and E4 mice after 14 days of recovery from barium chloride-induced muscle damage *in vivo*, and primary MuSCs were isolated to assess proliferation and differentiation of ApoE2, E3, and E4 MuSCs *in vitro*.

**Results:** There was no difference in the basal skeletal muscle phenotype of ApoE isoforms as evaluated by section area, myofiber cross-sectional area (CSA), and myonuclear and MuSC abundance per fiber. Although there were no differences in fiber-type frequency in the soleus, Type IIa relative frequency was significantly lower in plantaris muscles of ApoE4 mice compared to ApoE3. Moreover, ApoE isoform did not influence muscle regeneration as assessed by fiber frequency, fiber CSA, and myonuclear and MuSC abundance. Finally, there were no differences in the proliferative capacity or myogenic differentiation potential of MuSCs between any ApoE isoform.

**Discussion:** Collectively, these data indicate nominal effects of ApoE isoform on the ability of skeletal muscle to regenerate following injury or the *in vitro* MuSC phenotype.

## Introduction

The human *APOE* gene encodes the glycoprotein apolipoprotein E which functions as a high-affinity ligand facilitating the clearance of diverse lipoproteins from systemic circulation (Mahley, 1988; Wernette-Hammond et al., 1989). The *APOE* gene is polymorphic, existing as three common isoforms - ApoE2, ApoE3, and ApoE4. The *APOE3* allele is the most common genotype, with 80% of the population carrying this allele. The less common E2 and E4 alleles are carried by 5%–10% and 10%–15% of the population, respectively (Zannis et al., 1981; Kim et al., 2009). The difference between the ApoE isoforms is a single amino acid substitution that induces a structural change within the protein which alters the capacity to bind the low-density lipoprotein receptor and modifies lipid binding preferences (Pitas et al., 1987; Huang, 2010). These alterations cause divergent cellular metabolism and have substantial implication in Alzheimer’s disease risk (Pitas et al., 1987; Huang, 2010).

Prior studies have also investigated the impact of ApoE knockout (ApoE^−/−^) on skeletal muscle, where ApoE has been found to be concentrated at the neuromuscular junction (Akaaboune et al., 1994). In mouse models of hindlimb ischemia using ApoE^−/−^ mice, ApoE was reported to be necessary for mitochondrial function, macrophage infiltration, and muscle healing (Kang et al., 2008; Crawford et al., 2013; Lejay et al., 2019). Similarly, skeletal muscle regeneration is also impaired in ApoE^−/−^ mice following cardiotoxin injury (Barlow et al., 2021).

In adult skeletal muscle, muscle stem cells (MuSCs) typically reside in a quiescent state, but in response to a stimulus (physiological or pathological), MuSCs exist quiescence and become activated, proliferate, and differentiate into multinucleated myofibers (Snijders et al., 2015). The role of MuSCs in muscle fiber repair and remodeling in response to exercise has been widely investigated (Bazgir et al., 2017). A number of studies have reported an increase in MuSC content during prolonged resistance type exercise training as well as positive correlations between increases in muscle fiber size and MuSC content (Petrella et al., 2006; Verdijk et al., 2010; Mackey et al., 2011; Bellamy et al., 2014). Furthermore, evidence suggests that MuSCs may play a role in non-hypertrophic skeletal muscle remodeling in response to aerobic exercise training (Joanisse et al., 2013; Joanisse et al., 2015). Notably, MuSCs from ApoE^−/−^ mice show delayed activation and differentiation on single muscle fibers *ex vivo* and impaired proliferation and differentiation *in vitro* (Barlow et al., 2021). This suggests that compromised MuSC function in ApoE^−/−^ mice may play a role in the impaired muscle regeneration.

We previously reported that, relative to ApoE2, ApoE3 and ApoE4 cells have higher glycolytic flux with increased and decreased oxidative TCA activity, respectively (Williams et al., 2020). Given the importance of central carbon metabolism to MuSC function, we hypothesized that muscle regeneration will be altered in ApoE4 mice compared to ApoE2 and ApoE3 mice. To test this hypothesis, we assessed muscle regeneration of ‘humanized’ ApoE mice following muscle injury.

## Materials and methods

### Animals and experimental procedures

Six-month old C57BL/6 mice homozygous for either the human E2, E3, or E4 *APOE* allele, as described previously, were used in this study (Williams et al., 2020; Lee et al., 2023a; Lee et al., 2023b). Mice were provided with sterilized food and acidified water *ad libitum* and kept under barrier conditions in a dedicated room with constant temperature and a 14:10 light cycle. ApoE2, E3, and E4 mice (*n* = 3 per group) were used for basal skeletal muscle phenotype assessment via immunohistochemistry (IHC). Further groups of E2, E3, and E4 mice (*n* = four to five per group) were treated with BaCl_2_ unilaterally, as described in further detail below, for assessment of skeletal muscle regeneration. For *in vitro* experiments, myogenic progenitor cells (MPCs) were isolated from 12-month old E2, E3, or E4 mice (*n* = two to three per group). Prior to muscle collections, mice were euthanized by cervical dislocation following isoflurane anesthetization. All procedures were approved by the University of Kentucky’s Institutional Animal Care and Use Committee.

### Single-cell transcriptomic data analysis

Single-cell transcriptomic data of regenerating mouse muscle tissue were acquired via Gene Expression Omnibus (GEO) under accession numbers GSE159500 and GSE162172 (McKellar et al., 2021). Briefly, all datasets for Days 0 and 5 post-muscle damage were downloaded and integrated by Seurat Integration, with integration feature anchors set at 3,000 features (Hao et al., 2021). Unbiased clustering was performed using shared nearest neighbor (SNN) clustering, and the resolution for cluster determination (FindClusters function) was set at 1. Data was visualized through Dimplot (UMAP), Featureplot (*Pax7*, *Apoe*, *Acta1*), and VlnPlot (*Pax7*, *Apoe*, *Acta1*). Paired box 7 (*Pax7*), a gene that is highly expressed in quiescence and is rapidly downregulated upon activation, was used to identify the “Quiescent” cluster of MuSCs. Mature myocytes were classified based on expression of skeletal muscle actin alpha 1 (*Acta1*) (Barruet et al., 2020). All newly developed code will be available on Github upon publication.

### Barium chloride injections

ApoE2, E3, and E4 mice were treated with BaCl_2_ as previously described (Dungan et al., 2022). A power analysis based on this previous study (Dungan et al., 2022) indicated that n = 4 mice per group provides sufficient power (1-β = 0.8) to detect differences in muscle regeneration outcome measures. Briefly, 10 μL of 1.2% BaCl_2_ was unilaterally injected into 5 locations along the length of the tibialis anterior (TA) muscle. The contralateral leg was injected with phosphate-buffered saline (PBS) in the same manner, serving as the control. Mice were euthanized 14 days post-injection. The 14-day time point was chosen to assess regeneration during later tissue shaping stages of muscle injury following differentiation and fusion of most MuSCs (Cutler et al., 2022).

### Immunohistochemistry

For immunohistochemical analyses, TA, soleus, and plantaris muscles were carefully excised, weighed, pinned to an aluminum-covered cork block at resting length, covered in Tissue-Tek optimal cutting temperature (OCT) compound (Sakura Finetek, Torrance, CA, United States), flash-frozen in liquid nitrogen-cooled isopentane, and stored at −80°C. Fresh-frozen muscle tissue were later removed from −80°C storage, placed into a cryostat (HM525 NX; Thermo Fisher, Waltham, MA, United States) set at −24°C, and 7 μm sections were cut and allowed to dry for at least 1 h before staining.

For soleus and plantaris fiber-type staining, procedures were carried out as previous described (Murach et al., 2019). Briefly, unfixed sections (7 µm) were incubated with antibodies against myosin heavy chain (MHC) types I (BA.D5), IIA (SC.71), and IIB (BF.F3) (1:100; Developmental Studies Hybridoma Bank, Iowa City, IA), as well as rabbit anti-laminin IgG (1:100; L9393; Sigma-Aldrich, St. Louis, MO) at room temperature for 90 min. In order to visualize MHC and laminin expression, fluorescence-conjugated secondary antibodies were applied to different mouse immunoglobulin subtypes for 1 h. MHC type IIX expression was inferred from unstained fibers.

Pax7/laminin/4′,6-diamidino-2-phenylindole (DAPI) staining was carried out as previously described (Murach et al., 2020). In summary, sections were fixed in 4% paraformaldehyde (PFA) and incubated in 3% H_2_O_2_ for 10 min, followed by epitope retrieval using sodium citrate (10 mM, 6.5 pH) at 92°C for 20 min. Sections were blocked (2% BSA plus Mouse on Mouse detection kit.; Vector Labs, Newark, CA, United States) and incubated overnight with antibodies against Pax7 (1:100; PAX7; Developmental Studies Hybridoma Bank), as well as rabbit anti-laminin IgG (1:100; L9393; Sigma-Aldrich). For visualization of Pax7 and laminin, fluorescence-conjugated secondary antibodies were applied to different mouse immunoglobulin subtypes for 90 min.

Muscle sections were imaged using a Zeiss upright microscope (AxioImager M1, Zeiss, Oberkochen, Germany) at 20x magnification and analyzed by MyoVision software (Wen et al., 2018; Viggars et al., 2022).

### Primary myogenic progenitor cell (MPC) isolation

MPCs were isolated from the hindlimb musculature of ApoE2, E3, and E4 mice. Tissue was homogenized using the gentleMACS Octo Dissociator and Skeletal Muscle Dissociation Kit (Miltenyi Biotec, Bergisch-Gladbach, Germany) according to manufacturer’s instructions. The cell suspension was then centrifuged, resuspended in MACS buffer (Miltenyi Biotec), and subsequently incubated with Satellite Cell Isolation Kit (Miltenyi Biotec) for 15 min at 4°C. MPCs (or satellite cells/myoblasts) were isolated using magnetic separation with the autoMACS Pro Separator (Miltenyi Biotec) according to manufacturer’s directions.

### Cell culture

Primary MPCs were cultured on 10% Matrigel-coated Primaria culture plates (Corning Inc., Corning, NY, United States) in growth media consisting of Hams F-10 (#10-070-CV; Corning Inc.), 20% fetal bovine serum (FBS) (35-070-C; Corning Inc.), 1% penicillin-streptomycin (97063-708; VWR, Radnor, PA, United States), and 10 ng/mL basic fibroblast growth factor (354060; Corning Inc.).

### *In vitro* MPC proliferation

To assess proliferation, cells were split via trypsinization (L0154-0100; VWR) into 24-well plates (5,000 cells/well) and subsequently incubated with 10 μM 5-ethynyl-2′-deoxyuridine (EdU) for 3 h. After 3 h, cells were fixed with 4% PFA, permeabilized with 0.5% Triton-X, and stained using a Click-iT EdU Assay (Thermo Fisher Scientific), followed by DAPI staining for 15 min (1:1,000, D1306, Thermo Fisher Scientific). Cells were imaged using an Axio Observer 7 inverted microscope (Zeiss) at 20x magnification and analyzed using Zen Blue Software (Zeiss). Proliferation was assessed by measuring the ratio of EdU^+^/DAPI^+^ nuclei relative to the total number of DAPI^+^ nuclei. Measurements were calculated based on the average of 15 frames per sample.

### *In vitro* MPC differentiation

For differentiation into myotubes, MPCs were plated on CYTOOchips coverslips with micropatterns (Myogenesis FN, CYTOO, Cambridge, MA) (50,000 cells/chip). Growth media was exchanged for differentiation media consisting of DMEM (30-2006; ATCC, Manassas, VA) supplemented with 2% horse serum (HS) (35-030-CV; Corning), and cells were allowed to differentiate for 7 days before immunostaining. Cells were fixed with 4% PFA and incubated in primary antibody against MHC (MF 20) (1:100, Developmental Studies Hybridoma Bank) overnight at 4°C, followed by incubation in fluorescent secondary antibody and DAPI (1:1,000) for 1 h at room temperature. Cells were imaged using an Axio Observer 7 inverted microscope (Zeiss) or an Olympus BX61S Virtual Slide Microscope (Olympus NDT, Waltham, MA) at 20x magnification and analyzed using Zen Blue Software (Zeiss). Myotube diameter was calculated as the average of 5 randomly chosen myotubes per sample, and the fusion index was calculated as the number of nuclei incorporated into myotubes expressed as a percentage of the total number of nuclei in the image frame averaged based on 5 frames per sample.

### Statistical analyses

One-way ANOVAs with *post hoc* Šídák corrections for multiple comparisons were used to compare measures of skeletal muscle phenotype in the soleus and plantaris of E2, E3 and E4 mice. Differences in TA section area, cross-sectional area (CSA), myonuclei, and satellite cells in PBS and BaCl_2_ treated limbs between E2, E3, and E4 mice were analyzed using two-way ANOVAs with Tukey corrections for multiple comparisons. A two-way ANOVA controlled for false discovery rate using the Benjimini, Krieger, and Yekutieli correction was used to compare TA fiber type CSA distribution in E2, E3, and E4 mice. One-way ANOVAs with Tukey corrections for multiple comparisons were used to assess differences in measures of proliferation and differentiation between E2, E3, and E4 mice. All statistical analyses were performed using GraphPad Prism version 9.5.1 for Windows (GraphPad Software, La Jolla, CA).

## Results

### *Apoe* is highly expressed in quiescent MuSCs

**FIGURE 1:**
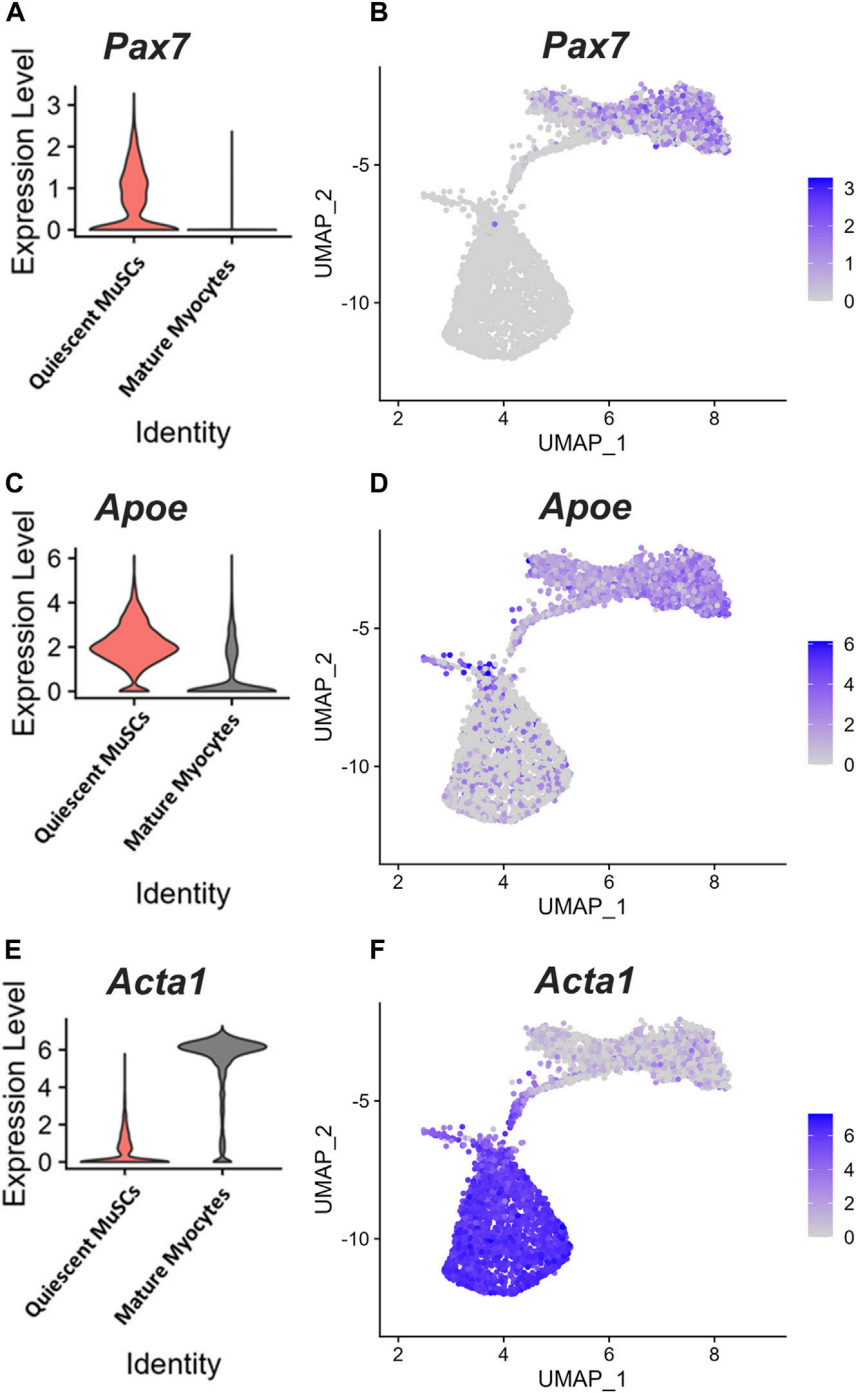
*Apoe* is highly expressed in quiescent MuSCs. Single-cell RNA-sequencing analysis of datasets generated from studies investigating skeletal muscle regeneration in mice. **(A)** Violin plot showing expression of *Pax7* in quiescent muscle stem cells (MuSCs) and mature myocytes, **(B)** Unbiased clustering and 2-dimensional uniform manifold approximation and projection (UMAP) representation of myogenic cells in muscle at days 0, 2, 5, and 7 following injury. Color scale represents *Pax7* expression level. **(C–F)** Violin plots and UMAP representation showing *Apoe* **(C, D)** and *Acta1* **(E, F)** expression levels.

*APOE* was identified by single-cell RNA-sequencing (scRNA-seq) as one of the top expressed genes in quiescent MuSCs (Barruet et al., 2020). Given this finding, we sought to assess the dynamics of *Apoe* expression during muscle regeneration. ScRNA-seq analysis of data generated from a study investigating skeletal muscle regeneration in mice (McKellar et al., 2021) confirmed that *Apoe* was one of the top expressed genes in quiescent MuSCs. UMAP unsupervised clustering to visualize the scRNA-seq data showed that clusters of myogenic cells characterized by high expression of *Pax7* (quiescent MuSCs) also expressed higher levels of *Apoe* (Figures 1A–D). In contrast, *Apoe* expression was substantially lower in mature myocytes with high *Acta1* expression (Figures 1E,F).

### ApoE isoform has nominal effects on adult skeletal muscle phenotype

**FIGURE 2:**
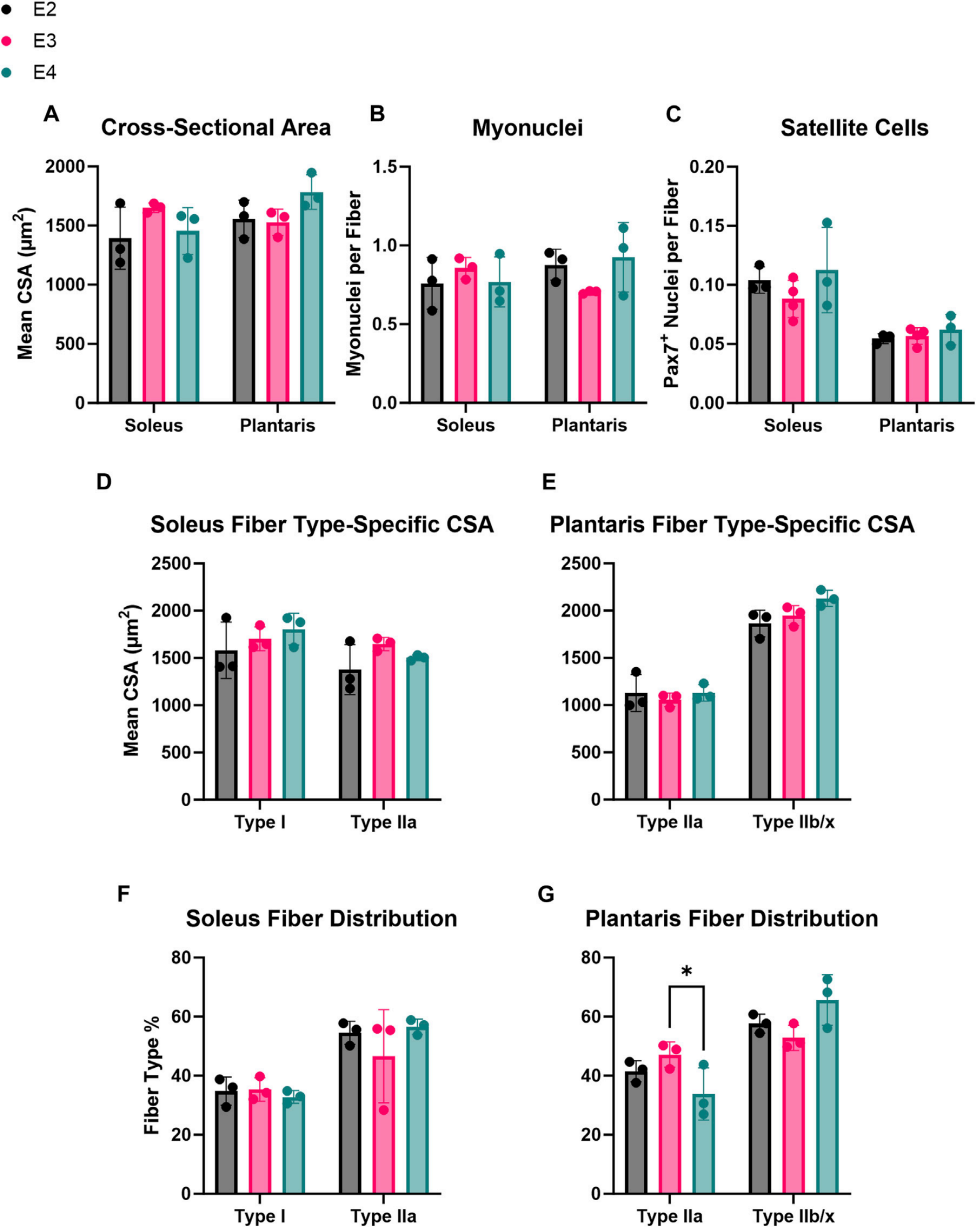
ApoE isoform induces minimal effects on adult skeletal muscle phenotype. Immunohistochemical analyses of basal skeletal muscle phenotype in E2, E3, and E4 mice. **(A)** Cross-sectional area (CSA), **(B)** number of myonuclei per fiber, and **(C)** number of satellite cells (MuSCs) per fiber in the soleus and plantaris of E2, E3, and E4 mice. **(D, E)** Fiber type distribution (% of total fibers) in the **(D)** soleus and **(E)** plantaris of E2, E3, and E4 mice. **(F, G)** Fiber-type specific CSA in the **(F)** soleus and **(G)** plantaris of E2, E3, and E4 mice.

To determine if the different human ApoE isoforms affected skeletal muscle phenotype under resting conditions, IHC was used to measure mean fiber CSA, fiber-type composition, and myonuclei and satellite cell abundance of the soleus and plantaris muscles from E2, E3, and E4 mice. There were no significant differences in mean CSA, fiber-type specific CSA, and myonuclear or satellite cell abundance per fiber between the E2, E3, and E4 groups for either the soleus or plantaris muscles (Figures 2A–E). Although there were no differences in fiber-type distribution in the soleus muscles (Figure 2F), plantaris Type IIa fiber relative frequency was significantly lower in ApoE4 mice compared to ApoE3 (*p* = 0.04) (Figure 2G). These data indicate that differing ApoE isoforms may induce only minimal changes in adult muscle phenotype under resting conditions.

### ApoE isoform does not affect skeletal muscle regeneration

**FIGURE 3:**
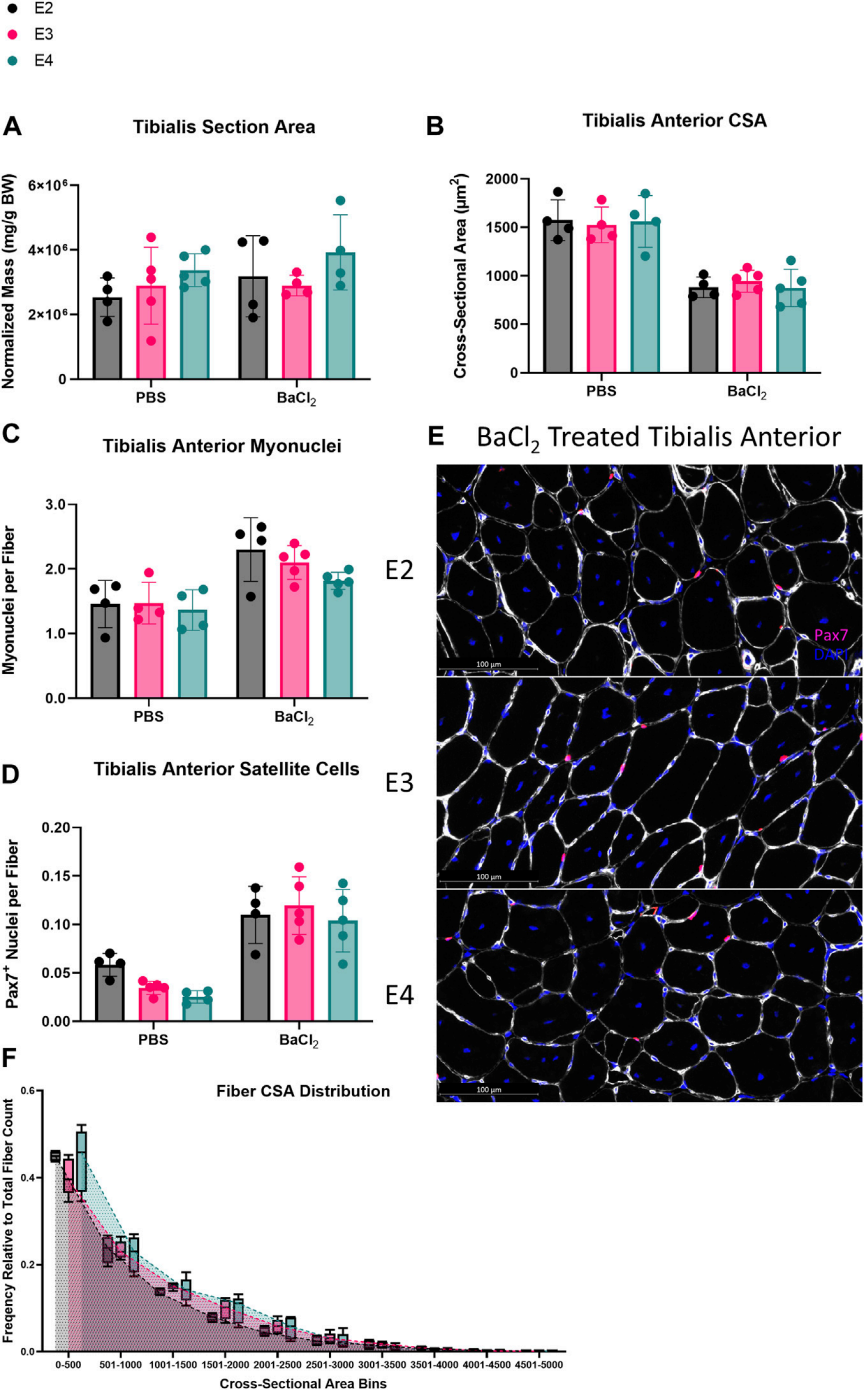
ApoE isoform does not affect skeletal muscle regeneration. Skeletal muscle regeneration in E2, E3, and E4 mice. **(A)** Total section area, **(B)** cross-sectional area (CSA), **(C)** myonuclei per fiber, and **(D)** satellite cells per fiber of PBS and barium chloride (BaCl_2_) treated tibialis anterior (TA) from E2, E3, and E4 mice. **(E)** Representative images of Pax7/laminin staining of BaCl_2_ treated TA cross-sections from E2, E3, and E4 mice. **(F)** Distribution of fiber CSA in 500 μm^2^ bins.

MuSCs undergo a metabolic shift towards glycolysis during activation and fusion (Ryall, 2013; Ryall et al., 2015; Pala et al., 2018; Yucel et al., 2019; Bhattacharya and Scime, 2020; Nalbandian et al., 2020). Thus, we hypothesized muscle regeneration would be enhanced in ApoE4 mice given that ApoE4-expressing cells have been shown to have a higher glycolytic flux than ApoE2 and ApoE3 cells (Farmer et al., 2021). After 14 days of muscle regeneration, there were no significant differences in TA total section area, mean fiber CSA, or the abundance of myonuclei or satellite cells per fiber between any of the ApoE isoform groups (Figures 3A–E). Fibers were grouped into 500 µm^2^ bins ranging from 0 to 5000 µm^2^. Posthoc analyses revealed no significant differences in fiber CSA distribution in any fiber CSA grouping between E2, E3 and E4 mice (Figure 3F). Taken together, these data demonstrate that ApoE isoform does not affect skeletal muscle regeneration when assessed at 14 days post-injury.

### ApoE isoform does not alter myogenic progenitor cell proliferation or differentiation *in vitro*

**FIGURE 4:**
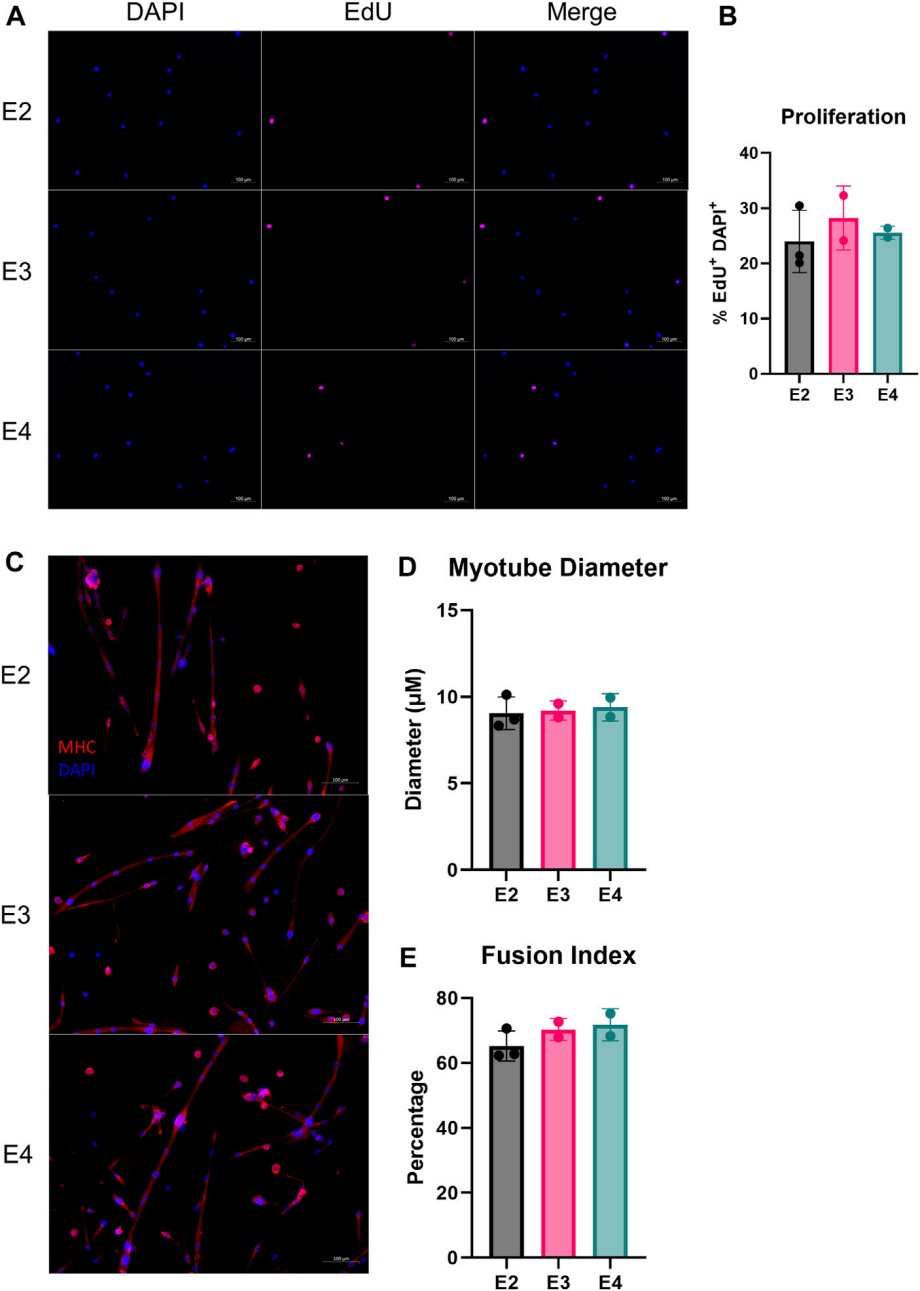
ApoE isoform does not alter myogenic progenitor cell proliferation or differentiation *in vitro*. Proliferation and differentiation of muscle stem cells (MuSCs) of E2, E3, and E4 mice. **(A)** Representative images of DAPI and EdU stained primary MuSCs and **(B)** proliferative capacity, assessed as the percentage of DAPI^+^/EdU^+^ cells relative to total DAPI^+^ cells from E2, E3, and E4 mice. **(C)** Representative images of differentiated myofibers plated on CYTOOchips coverslips and stained for myosin heavy chain (MHC) and DAPI. **(D)** Myotube diameter and **(E)** the percentage of myotube-associated nuclei relative to total nuclei (fusion index) after 7 days of differentiation of primary MuSCs isolated from E2, E3, and E4 mice.

Next, we measured myogenic progenitor cell (MPC) proliferation and differentiation capabilities of the different ApoE isoforms. First, we isolated MPCs from ApoE2, E3, and E4 mice and measured EdU incorporation *in vitro*. We did not observe a significant difference in the percentage of EdU^+^ cells between ApoE isoform groups (Figures 4A,B), indicating the rate of MPC proliferation is similar between groups. We next assessed the ability of MPCs to undergo myogenic differentiation. Based on myotube diameter and the fusion index, we did not observe any difference in differentiation between ApoE2, E3, and E4 mice (Figures 4C–E).

## Discussion

The present study aimed to investigate the effects of ApoE isoforms in modulating MuSC behavior in response to muscle damage *in vivo*. MuSCs have been shown to primarily utilize fatty acid oxidation during quiescence, shifting their metabolism to glycolysis upon activation (Ryall, 2013; Ryall et al., 2015). Interestingly, ApoE4 microglia (Lee et al., 2023a) and astrocytes (Williams et al., 2020; Farmer et al., 2021) have increased glycolysis and reduced fatty acid metabolism. Thus, it is plausible to speculate that ApoE4 mice may be primed for rapid activation following muscle damage. However, following BaCl_2_-induced damage, we found no differences between ApoE2, ApoE3, or ApoE4 mice in muscle fiber CSA, myonuclear density, or MuSC number.

To better isolate mechanistic differences of the different ApoE isoforms on MuSC biology, we isolated primary MuSCs from E2, E3, and E4 mice. *In vitro*, there were no differences in the proliferative capacity or myogenic differentiation potential between MuSCs expressing any ApoE isoform. Thus, in contrast to microglia and astrocytes, there may be no difference in MuSC metabolism between different ApoE isoforms. Furthermore, basal metabolism of MuSCs may not play a major role in muscle regeneration since MuSCs undergo extensive metabolic alterations during myogenesis (Joseph and Doles, 2021). Differing ApoE isoforms may not impact the MuSC metabolic switch to glycolysis that occurs upon activation, especially considering the downregulation in ApoE that occurs simultaneously in activated MuSCs. However, future studies should assess the oxygen consumption and extracellular acidification rates of ApoE2, ApoE3, and ApoE4 MuSCs to better characterize metabolic properties associated with the different isoforms.

As revealed by our analysis of scRNA-seq data, *APOE* expression is highest in quiescent MuSCs, and *APOE* expression is downregulated in mature myocytes following activation and differentiation. In light of these findings, future research endeavors should focus on investigating ApoE isoform-specific effects in the quiescent MuSC state when ApoE variant disparities may be most prominent. Further studies should also investigate ApoE isoform-specific differences in MuSC signaling and response to signaling molecules. ApoE signaling, which can involve both direct binding to ApoE receptors as well as interactions with other receptors such as N-methyl-D-aspartate receptors, may modulate MuSC quiescence, a state controlled by various cell adhesion molecules and signaling pathways (Montarras et al., 2013; Lane-Donovan and Herz, 2017; Zhou et al., 2021).

It is important to note that our sample size for some of the analyses included was limited, which may have reduced the power to identify significant differences between groups. However, for the primary outcomes associated with the muscle regeneration study, the experiment was sufficiently powered. It is worth acknowledging that in the current study, we utilized adult mice. ApoE levels are known to increase with age in several tissues, and ApoE is thought to modify aging and homeostatic adaptive responses during aging (Yassine and Finch, 2020; Zhao et al., 2022). Thus, we cannot rule out the possibility that aged mice expressing the different ApoE isoforms may demonstrate differing muscle regenerative capacities. Furthermore, although we found no differences in basal muscle characteristics between ApoE2, ApoE3, and ApoE4 young mice, life-long expression of the different ApoE variants may result in differing skeletal muscle phenotypes in aged mice.

Finally, chemical injury using barium chloride is a non-physiological model of muscle regeneration. While this experimental model can provide proof of principle for MuSC biological phenomena, the mechanisms can be different from physiological repair that occurs in response to exercise-induced skeletal muscle adaptation, which results in a far more subtle degree of muscle damage (Fukada et al., 2022). In fact, in response to barium chloride, the MuSC population is almost entirely destroyed along with the muscle fibers (Murach et al., 2021). Furthermore, functions of MuSCs in the context of exercise adaptation that are independent from myonuclear addition have been proposed (Murach et al., 2020). Thus, while we found that muscle regeneration was not affected by ApoE isoform under extreme muscle damage, future studies should also evaluate whether exercise-induced muscle damage recovery or adaptation is influenced by ApoE isoform.

## Data Availability

Single-cell transcriptomic data of regenerating mouse muscle tissue were acquired via Gene Expression Omnibus (GEO) under accession numbers GSE159500 and GSE162172.
